# Fermented *Angelicae tenussimae* with *Aspergillus oryzae* Improves Skin Barrier Properties, Moisturizing, and Anti-Inflammatory Responses

**DOI:** 10.3390/ijms232012072

**Published:** 2022-10-11

**Authors:** Chang-Woo Ha, Eun-Hwa Sohn, Sung-Hyeok Kim, Sohee Jang, Myung-Rye Park, Youn-Kyu Kim, In-Young Bae

**Affiliations:** 1 Department of Bio-Health Convergence, Kangwon National University, Chuncheon 24341, Korea; 2 Korea Research Institute Bio Science Co., Ltd., Yeoju 12668, Korea; 3 Department of Food and Nutrition, Far East University, Eumseong 27601, Korea

**Keywords:** *Angelicae tenussimae*, ligustilide, atopic dermatitis, skin barrier, moisturizing, inflammation

## Abstract

*Angelicae tenussimae* root has been used as a traditional medicine in Asia. Recently, anti-melanogenic and anti-photogenic effects of fermented *A. tenuissima* root (FAT) were identified. However, information about the anti-atopic dermatitis action of FAT is limited. Thus, the purpose of this study is to determine the applicability of FAT to AD by identifying the efficacy of FAT on the skin barrier and inflammatory response, which are the main pathogenesis of AD. Expression levels of skin barrier components and the production of inflammatory mediators in human keratinocyte and mouse macrophage cells were measured by quantitative RT-PCR or ELISA. FAT upregulated the expression of skin barrier components (filaggrin, involucrin, loricurin, SPTLC1) and inhibited the secretion of an inflammatory chemokine TARC in HaCaT cells. Furthermore, it suppressed pro-inflammatory cytokines (IL-6, TNF-α) and nitric oxide production in LPS-induced RAW264.7 cells. In addition, ligustilide increased filaggrin and SPTLC1, and also lowered pro-inflammatory mediators that increased in atopic environments, such as in FAT results. This means that ligustilide, one of the active ingredients derived from FAT, can ameliorate AD, at least in part, by promoting skin barrier formation and downregulating inflammatory mediators. These results suggest that FAT is a potential functional cosmetic material for the care and management of AD.

## 1. Introduction

Atopic dermatitis, a more common disease in Westernized countries, has a prevalence of 0.96~22.6% in children and 1.2~17.1% in adults, and has recently shown a sharp increase in Korea [1]. Atopic dermatitis is a chronic inflammatory skin disease characterized by dryness, predisposition, and erythema eczema, which are often accompanied by other atopic diseases such as allergic rhinitis and asthma [2]. Currently, it was revealed that the onset of atopic dermatitis was due to skin barrier damage; additionally, skin barrier dysfunction and abnormal immune responses were the main causes of atopic dermatitis [3]. The stratum corneum mainly composed of corneodesmosome and corneocyte is the outer layer of the skin, preventing water loss in the body and invading external harmful substances [2]. The cornified envelope protein serves as a physical barrier, which is formed through cross-linking of proteins such as involucrin, loricrin, trichohyalin, and small proline-rich proteins [4]. Filaggrin, which plays an important role in creating brick structures in the skin barrier by acting as an adhesive that binds keratins to each other, has recently attracted attention as one of the genetic factors of atopic dermatitis and its moisturizing effect in retaining water [5]. Amino acids such as pyrrocarboxylic acid (PCA) and trans-urocanic acid (UCA), which are the final degradation products of filaggrin, act as natural moisturizing factors [6]. Besides the cornified envelope protein, the changes in the cytokines involved in the formation of skin barriers can lead to skin barrier abnormalities in inflammatory skin diseases [7]. Furthermore, the expression of inflammatory cytokine and pruritus factors such as thymus and activation-regulated chemokine (TARC/CCL17, TARC) in the epidermis increases by acute damage to the skin barrier, which can negatively affect the composition and function of the skin barrier and the normal epidermal-differentiation process. TARC is a Th2-associated chemokine produced by keratinocytes that recruits lymphocytes to atopic dermatitis lesions. Monitoring serum TARC concentration during intervention for atopic dermatitis is valuable for determining disease activity and evaluating therapeutic efficacy, so TARC level is also considered a clinical biomarker of atopic dermatitis severity [8]. It is also important to suppress inflammatory factors along with improving skin barriers and moisturizing factors to improve the quality of care in atopic dermatitis. Since atopic skin requires treatment and management at the same time, it is very necessary to develop safe natural products with few side effects that can be used for a long time.

*Angelica tenuissima* Nakai (*A*. *tenuissima*) is one of the traditional Asian medicines that has been used to treat headaches, diarrhea, epilepsy, rheumatic arthritis, and gynecological diseases and anemia for women [9]. Recently, it has been demonstrated to have various anti-viral, anti-osteoporosis, and anti-inflammatory effects [10,11,12]. In our previous study, it was confirmed that fermented *A. tenuissima* root (FAT) had an anti-melanogenetic effect, and we found that the content of ligustilide, and one of the active compounds, was higher during fermentation by *Aspergillus oryzae* [13,14]. However, the effect of FAT and ligustilide on the skin barrier, the outermost layer of first line of defense, has not been reported yet. To further expand the efficacy studies on FAT and apply them to the care and maintenance of atopic skin, we investigated the effects of FAT on skin barrier properties and inflammatory factors. First, the expression level of skin barrier components (filaggrin, involucrin, and loricurin) in human keratinocyte was determined by quantitative RT-PCR (qPCR). Second, the expression levels of serine-palmitoyltransferase (SPT; a key enzyme for the initial step of ceramide biosynthesis) and TARC, a typical type 2 helper T cell-secreted chemokines, were measured with qPCR. Finally, the production of pro-inflammatory mediators (IL-6, TNF-α, and NO) was examined in LPS-induced mouse macrophage cells by RT-PCR or ELISA analysis. In addition, the effects of ligustilide as an active component of FAT on skin barrier properties and inflammatory factor suppression were elucidated. These results suggested that FAT could be a functional cosmetic material for the care and the management of atopic dermatitis.

## 2. Results

### 2.1. Effects of FAT and Ligustilide on Cell Viability

**Figure 1:**
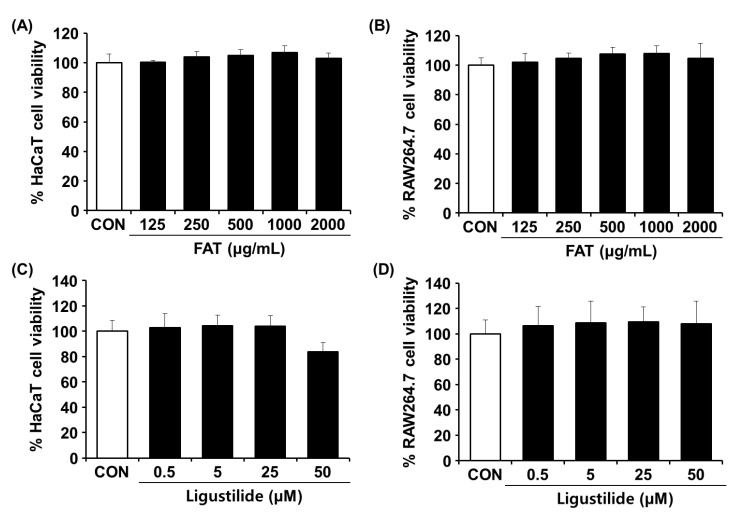
Cytotoxic effects of FAT and ligustilide in HaCaT and RAW264.7 cells. (**A**) HaCaT and (**B**) RAW264.7 cells were treated with FAT (125~2000 ug/mL) for 24 h. (**C**) HaCaT and (**D**) RAW264.7 cells were treated with ligustilide (0.5~50 uM) for 24 h. Cell viability was performed by MTT assay. Data represent the mean ± SD of three independent experiments. Statistical significance was assessed by one-way ANOVA followed by Tukey’s test. FAT; Fermented *Angelicae tenussimae* with *Aspergillus oryzae*.

The cytotoxicity of FAT and ligustilide to HaCaT and RAW264.7 cells was measured using an MTT assay at concentration ranges of 125~2000 ug/mL and 0.5~50 uM, respectively. After FAT treatment for 24 h, it was observed that FAT did not show a significant cytotoxic effect at any concentrations used (Figure 1A,B). Therefore, the experiment was performed with FAT at a concentration range of 125~1000 ug/mL. After ligustilide treatment for 24 h, 16.2% of cytotoxicity was observed at 50 uM of ligustilide in HaCaT cells, and no cytotoxicity was observed in RAW264.7 cells at all treated concentrations (Figure 1C,D). Therefore, the experiment was performed in a concentration range of 0.5–50 uM, which did not show significant cytotoxicity in both cells.

### 2.2. Effects of FAT on mRNA Expression of Filaggrin, Involucrin, and Loricurin in HaCaT Cells

**Figure 2:**
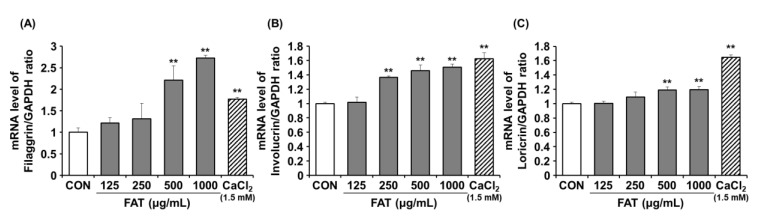
Effects of FAT on the mRNA expression of filaggrin, involucrin, and loricurin in HaCaT cells. (**A**) Filaggrin, (**B**) involucrin, and (**C**) loricurin were determined by RT-PCR. Data represent the mean ± SD of three independent experiments. Statistical significance was assessed by one-way ANOVA followed by Tukey’s test. ** *p* < 0.01 compared with the untreated cells. FAT; Fermented *Angelicae tenussimae* with *Aspergillus oryzae*.

We initially investigated the effects of FAT on expression levels of cornified envelope proteins such as filaggrin, involucrin, and loricurin in human keratinocytes. FAT increased mRNA expression levels of filaggrin, involucrin, and loricurin by 2.7-fold, 1.5-fold, and 1.2-fold at the dose of 1000 ug/mL, respectively, compared with the control (no FAT treatment) (Figure 2). Moreover, FAT at concentrations higher than 500 mg/mL significantly increased filaggrin mRNA levels, higher than the level increased by CaCl_2_ (1.5 mM) used as a positive control.

### 2.3. Effects of FAT on mRNA Expression of SPT and TARC in HaCaT Cells

**Figure 3:**
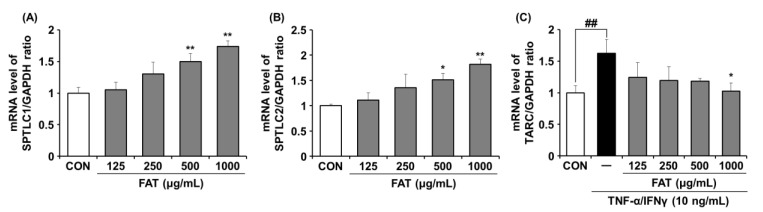
Effects of FAT on the mRNA expression of SPT and TARC in HaCaT cells. (**A**) SPTLC1, (**B**) SPTLC2, and (**C**) TARC were determined by RT-PCR. Data represent the mean ± SD of three independent experiments. Statistical significance was assessed by one-way ANOVA followed by Tukey’s test. * *p* < 0.05, ** *p* < 0.01, and ^##^ *p* < 0.01 compared with the untreated cells and control, respectively. compared with the untreated cells. FAT; Fermented *Angelicae tenussimae* with *Aspergillus oryzae*, SPTLC1; Serine Palmitoyltransferase Long Chain Base Subunit 1, SPTLC2; Serine Palmitoyltransferase Long Chain Base Subunit 2, TARC; Thymus and activation-regulated chemokine, TNF-α; Tumor necrosis factor α, IFN-γ; Interferon γ.

We next examined the effects of FAT on expression levels of SPT and TARC, which are known to play an important role in ceramide biosynthesis and immune regulation, respectively. The mRNA levels of Serine Palmitoyltransferase Long Chain Base Subunit 1 (SPTLC1) and SPTLC2 were higher in the FAT group than those in the untreated group of cells. At a concentration of 1000 ug/mL, FAT significantly increased mRNA levels of SPTLC1 and SPTLC2 by 1.7-fold and 1.8-fold, respectively (Figure 3A,B). Meanwhile, the TARC mRNA level was higher in TNF-α/IFNγ-stimulated HaCaT cells than in control cells (Figure 3C). However, treatment with FAT suppressed TARC expression dose-dependently. At a concentration of 1000 ug/mL, FAT significantly decreased TARC expression by 1.6-fold.

### 2.4. Effects of FAT on IL-6 Expression and Pro-Inflammatory Mediator Production in LPS-Induced RAW264.7 Cells

**Figure 4:**
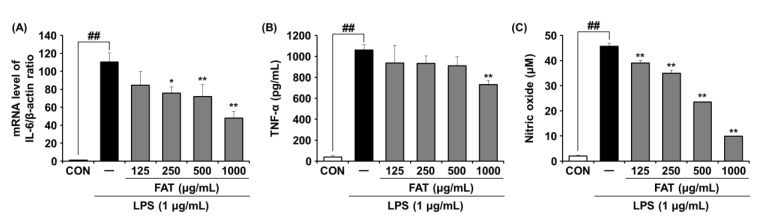
Effects of FAT on the mRNA expression of IL-6 and the pro-inflammatory mediator production in LPS-induced RAW264.7 cells. (**A**) IL-6 was determined by RT-PCR. (**B**) TNF-α and (**C**) NO production were measured by using ELISA. Data represent the mean ± SD of three independent experiments. Statistical significance was assessed by one-way ANOVA followed by Tukey’s test. * *p* < 0.05, ** *p* < 0.01, and ^##^ *p* < 0.01 compared with the untreated cells and control, respectively. FAT; Fermented *Angelicae tenussimae* with *Aspergillus oryzae*, IL-6; Interleukin 6, TNF-α; Tumor necrosis factor α, NO; Nitric oxide, LPS; Lipopolysaccharide.

We also performed RT-PCR to determine mRNA levels of IL-6 in LPS-induced mouse macrophage cells. It was found that IL-6 mRNA expression was higher in LPS-stimulated cells than in control cells. However, it was markedly lowered by FAT in a dose-dependent manner. FAT at 1000 ug/mL lowered the expression level of IL-6 by 2.3-fold (Figure 4A).

In addition, production levels of TNF-α and NO in LPS-induced mouse macrophage cells were measured using ELISA kits. Although amounts of TNF-α and NO were increased in LPS-induced cells than in control cells, they were significantly decreased when FAT was used to treat cells (Figure 4B,C). FAT at 1000 ug/mL notably reduced production levels of TNF-α and NO by 1.4-fold and 4.6-fold, respectively.

### 2.5. Effects of Ligustilide on mRNA Expression Levels of Filaggrin and SPTLC1 and Production of TARC in HaCaT Cells

**Figure 5:**
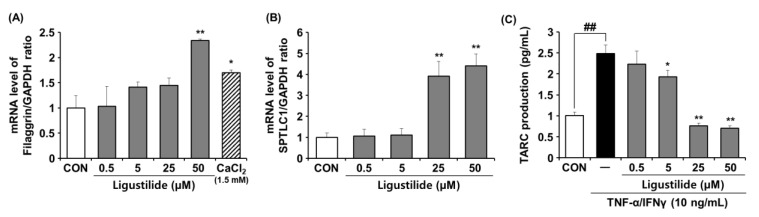
Effects of ligustilide on the mRNA expression of filaggrin and SPTLC1 and production of TARC in HaCaT cells. (**A**) Filaggrin and (**B**) SPTLC1 were determined by RT-PCR. (**C**) TARC production was measured by using ELISA. Data represent the mean ± SD of three independent experiments. Statistical significance was assessed by one-way ANOVA followed by Tukey’s test. * *p* < 0.05, ** *p* < 0.01, and ^##^ *p* < 0.01 compared with the untreated cells and control, respectively. SPTLC1; Serine-palmitoyltransferase, TARC; Thymus and activation-regulated chemokine, TNF-α; Tumor necrosis factor α, IFN-γ; Interferon γ.

To further elucidate the effect of FAT on skin barrier properties, we analyzed expression levels of filaggrin and SPT in human keratinocytes after treatment with ligustilide. In addition, the effect of ligustilide on TARC production, which plays an important role in immunomodulation, was investigated. As shown in Figure 5A, ligustilide treatment showed a 2.3-fold significant increase in filaggrin expression at a concentration of 50 uM. In addition, ligustilide treatment significantly increased SPTLC1 expression by 3.9-fold and 4.4-fold at 25 uM and 50 uM concentrations (Figure 5B). In the TARC experiment, ligustilde at concentrations of 20 uM and 50 uM significantly inhibited TNF-α/IFNγ-induced TARC production by 69% and 71% (Figure 5C). This is consistent with the result of ligustilde showing a large effect on SPTLC1 expression in the range of 20 uM and 50 uM. This means that the effective concentration of ligustild (about 20 uM or more) can exert a more beneficial effect on skin barrier improvement.

### 2.6. Effects of Ligustilide on IL-6 Expression and Pro-Inflammatory Mediator Production in LPS-Induced RAW264.7 Cells

**Figure 6:**
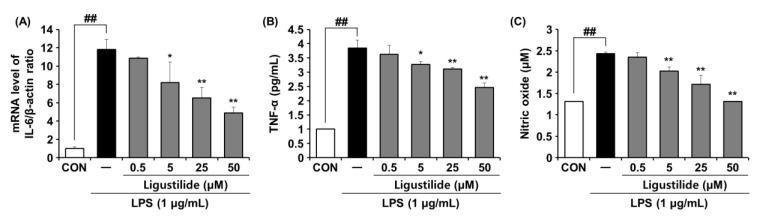
Effects of ligustilide on the mRNA expression of IL-6 and pro-inflammatory mediator production in LPS-induced RAW264.7 cells. (**A**) IL-6 was determined by RT-PCR. (**B**) TNF-α and (**C**) NO production were measured by using ELISA. Statistical significance was assessed by one-way ANOVA followed by Tukey’s test. * *p* < 0.05, ** *p* < 0.01 and ^##^ *p* < 0.01 compared with the untreated cells. TNF-α; Tumor necrosis factor α, NO; Nitric oxide, LPS; Lipopolysaccharide.

Finally, ligustilide was used to treat LPS-induced RAW264.7 cells. Production levels of pro-inflammatory mediators (IL-6, TNF-α, and NO) were then measured using qPCR or ELISA. When cells were treated with ligustilide, the increases in IL-6, TNF-α and NO by LPS, an inflammatory response inducer, were significantly reduced at all treatment concentrations except 0.5 nM (Figure 6). This means that ligustilide is a potential ingredient with anti-inflammatory effects.

## 3. Discussion

The potential of FAT as a functional cosmetic agent has been identified in melanogenic and photogenic conditions [13,14,15]. However, the alleviating effect of FAT against atopic dermatitis has not been reported yet. In this study, we found that FAT dose-dependently increased mRNA levels of cornified envelope proteins (filaggrin: 2.7-fold; loricurin: 1.5-fold; involucrin: 1.2-fold) and SPTs (SPTLC1: 1.7-fold; SPTLC2: 1.8-fold) in HaCaT cells. Furthermore, FAT decreased the production of TARC (1.6-fold) in TNF-α/IFNγ-stimulated HaCaT cells and pro-inflammatory mediators (IL-6: 2.3-fold; TNF-α: 1.4-fold; NO: 4.6-fold) in LPS-stimulated RAW264.7 cells. These results suggest that FAT shows its anti-atopic effect by enhancing the skin barrier formation and water retention and by regulating the secretion of pro-inflammatory mediators.

The skin is the most external organ of the human body. It functions as an essential barrier to protect the loss of moisture in the body and internal intrusion of external harmful factors such as antigen, microorganism, and source of infection. Atopic dermatitis, caused by hypersensitivity to harmless external antigens, is a chronic inflammatory disease due to altered skin barrier and immune dysregulation [16]. The skin barrier refers to the stratum corneum of the epidermis. It is described by the “brick and mortar” theory [17,18,19]. “Brick” describes keratinocytes with various proteins that make up the and membranes connected to them. Corneodesmosome connects keratinocytes. It is involved in the support and periodic elimination of keratinocytes. “Mortar” refers to intercellular lipids with multilayer structures mainly composed of ceramide, cholesterol, and fatty acids.

Filaggrin, which connects cornified envelope and keratin intermediate microfilament, plays an important role in building brick structures of skin barriers [5]. Decreased filaggrin production results in weakened keratinocyte membrane formation and reduced adhesion between keratinocytes, finally increasing transepidermal water loss. As a result, sensitization and pruritus easily occur due to increased allergen penetration because of alterations in physical skin barrier. In terms of “Mortar”, SPT is effective in maintaining moisture and strengthening skin barrier functions by increasing ceramide synthesis in keratinocyte [20,21]. Ceramide is an important ingredient that accounts for 50~60% of lipid components of the stratum corneum. It plays a critical role in maintaining moisture in the skin [22]. When the function of the skin barrier is impaired, ceramide is clearly reduced, which causes skin drying. In addition, decreased production of sphingosine, a ceramide-degrading product with antibacterial ability, is associated with skin barrier dysfunction due to increased allergic stimuli and exposure to bacteria and viruses. Yang et al. have shown that Lava-Dermabiotics HDB1234 can enhance moisture retention of the skin by increasing the expression of filaggrin and SPT in a dose-dependent manner [23]. In this study, we found that FAT could increase the expression of filaggrin, involucrin, loricurin, and SPT, showing a potential to protect skin barriers. 

TARC is one of the biomarkers that can identify immune abnormalities. It binds to CCR4 (CC chemokine receptor 4-positive) of Th2 cells. Th2 cells then migrate to the site of inflammation [24]. In the skin exposed to allergens due to skin barrier damage, a hyperimmune reaction occurs and pro-inflammatory chemokines such as TARC are produced in keratinocytes. Increased TARC can activates Th2 cells to promote the production of pruritus-causing IL-31 and induce the skin to an inflammatory chronic stage. Meanwhile, macrophages are distributed in all tissues of animals to digest invading bacteria. Activated macrophages can recognize the cause of inflammation and produce inflammatory mediators (such as prostaglandin and NO) and proinflammatory cytokines (such as TNF-α, IL-1, and IL-6) [25]. Therefore, it is important to suppress the production of pro-inflammatory mediators in order to prevent pruritus and inflammatory reactions. A previous study has reported that a novel *Sporichthyaceae* bacterium strain K-07 isolated from healthy human skin can change mRNA expression levels of filaggrin, IL-6, TNF-α, and TARC [26]. These results suggest that metabolite of strain K-07 can ameliorate skin barrier and anti-inflammatory effects. In addition, anti-atopic effects of mixed extracts from date plum, persimmon, and mulberry leaves (DPME) have been suggested from the perspective of natural anti-inflammatory ingredients [27]. DPME can reduce the production of pro-inflammatory mediators (NO, TNF-α, and IL-6) in LPS-stimulated RAW264.7 macrophages. Our study revealed that the expression level of TARC in TNF-α/IFNγ-stimulated HaCaT cells was decreased in the presence of FAT. In addition, amounts of pro-inflammatory mediators such as IL-6, TNF-α, and NO in LPS-induced RAW264.7 cells were decreased by FAT.

To determine which components contributed to the effect of FAT on atopic dermatitis treatment, ligustilide was used in the experiment. Ligustilide increased filaggrin and SPTLC1 in HaCaT cells in the same manner as in FAT. In addition, ligustilide inhibited the increased production of TARC, IL-6, TNF-α, and NO by TNF-α/IFNγ and/or LPS in a similar pattern as the FAT results. This means that ligustilide, one of the active ingredients derived from FAT, can ameliorate atopic dermatitis, at least in part, by promoting skin barrier formation, retaining water and downregulating inflammatory mediators.

In conclusion, (1) FAT is responsible for the production of structural proteins and ceramides constituting the skin barrier because it can increase the synthesis of filaggrin, involucrin, loricrin, and ceramide synthesis by upregulating SPT expression. In relation to immune imbalance (allergy and inflammatory reactions) and pruritus caused by skin barrier damage, (2) FAT is beneficial due to its anti-inflammation effect by inhibiting TARC overexpression and the production of inflammatory substances (IL-6, TNF-α, NO). Despite the identification of its underlying mechanisms for skin barrier improvement, the anti-atopic effect of FAT and ligustilide requires further study. Our observations suggest that FAT and its component ligustilide are potential functional cosmetic materials for the care and management of atopic dermatitis.

## 4. Materials and Methods

### 4.1. Materials

DMEM (Dulbecco’s modified eagle medium) and PBS (Phosphate buffer saline) were purchased from WelGENE (Daegu, Korea). FBS (Fetal bovine serum) and LPS (Lipopolysaccharide) were obtained from GenDepot (Katy, TX, USA) and Invitrogen (Thermo Fisher Scientific, Cleveland, OH, USA), respectively. Ligustilide, MTT (3-(4,5-dimethyl-thiazol-2-yl)-2,5 dephenyl-2H-tetrazolium bromide), DMSO (Dimethyl sulfoxide), CaCl_2_, IFN-γ (Interferon γ), TNF-α (Tumor necrosis factor α), NaNO_2_, Griess reagent, and other chemicals were produced from Sigma-Aldrich (St. Louis, MO, USA). The primers for quantitative RT-PCR (Table 1) were prepared by NanoHelix (Daejeon, Korea).

### 4.2. Preparation of Fermented Angelicae tenussimae with Aspergillus oryzae (FAT)

FAT was prepared as described previously [13,14]. Briefly, dried *A. tenuissima* roots were extracted with 70% ethanol at 80 °C for 4 h. The filtrate was concentrated under vacuum at 50 °C to 53 Brix. For the preparation of FAT, 40 mL of *A. tenuissima* root extract (20%, weight/volume) was added to 2 L of LB (Luria-Bertani) medium with 100 mL of seed culture (LB medium with *A. oryzae* incubating at 37 °C for 48 h) and then incubated at 37 °C for 48 h with shaking at 50 rpm. The fermented broth was subjected to extraction with ethanol (1:1 volume ratio) at 80 °C for 4 h, and centrifuged at 2000× *g* for 20 min at 4 °C. The supernatant was concentrated and lyophilized.

### 4.3. Cell Culture

Human keratinocyte HaCaT and mouse macrophage RAW264.7 were obtained from Cell Lines Service GmbH (Eppelheim, Germany) and Korean Cell Line Bank (Seoul, Korea), respectively. These cells were grown in DMEM supplemented with 10% FBS, penicillin (100 units/mL), and streptomycin (100 ug/mL) at 37 °C in a humidified atmosphere of 5% CO_2_. 

### 4.4. Cell Viability

Cells were seeded into 96-well plates at a density of 1 × 10^4^ cells/well (100 uL/well) and incubated culture medium at 37 °C for 24 h with 5% CO_2_. After treatment with FAT (125~2000 ug/mL) or ligustilide (0.5~50 uM), cells were further incubated for 24 h. MTT solution (5 mg/mL; 100 uL/well) was added and incubated for 4 h until formazan was formed. The formazan was dissolved in DMSO. The absorbance was then measured at 540 nm using a multi-detector microplate reader VICTOR X3 (PerkinElmer, Waltham, MA, USA).

### 4.5. RT-PCR Analysis

HaCaT and RAW264.7 cells were seeded into a 6-well plate at a density of 4 × 10^5^ cells/well and incubated 37 °C with 5% CO_2_ for 24 h. After washing with PBS, samples of 2 mL/well (including TNF-α/IFNγ (10 ng/mL) for TARC analysis) were added to cells and then cultured at 37 °C for 24 h with 5% CO_2_. Cultured cells were recovered, dissolved in 1 mL of triazole, and added 200 uL of chloroform. After centrifugation for 12 min, the supernatant was taken separately. Isopropanol (500 uL) was added to the supernatant. After centrifuging for 25 min, the pellet was obtained. After adding 80% ethanol, it was centrifuged for 5 min. The air-dried pellets were then suspended with diethyl pyrocarbonate-distilled water (DEPC-DW). The extracted RNA was quantified using a spectrophotometer. cDNA synthesis was performed with a cDNA synthesis kit (ECDNA100, NanoHelix, Deajeon, Korea) using the following reverse transcription conditions: 65 °C for 5 min, 42 °C for 10 min, 50 °C for 50 min, 70 °C for 10 min. The synthesized cDNA was subjected to PCR amplification using a premier RT-PCR kit (PQL-S500, NanoHelix, Deajeon, Korea). PCR amplification was performed at 95 °C for 30 s, followed by 39 cycles of 95 °C 5 s and 60 °C 30 s with a CFX connect Real-Time PCR detection system (BIO-RAD, Hercules, CA, USA).

### 4.6. TARC Production

HaCaT cells were pretreated with ligustilide for 1 h, and then stimulated with TNF-α (10 ng/mL) and IFNγ (10 ng/mL) for 20 h. Supernatants were collected and analyzed for TARC production by ELISA kits (R&D Systems, Minneapolis, MN, USA), according to the manufacturer’s instructions.

### 4.7. TNF-α and Nitric Oxide Assay

RAW264.7 cells were seeded into a 6-well plate at 4 × 10^5^ cells/well and incubated for 24 h under cell culture condition of 37 °C and 5% CO_2_. After washing with PBS, samples and LPS (1 ug/mL) were used to treat cultured cells. After incubation at 37 °C for 24 h with 5% CO_2_. Levels of TNF-α were measured using an ELISA MAX™ Deluxe Set Mouse TNF-α (Biolegend, San Diego, CA, USA). Nitric oxide (NO) production in the cell culture medium was determined with the Griess reaction. Briefly, the cultured cell medium was mixed with the same volume of Griess reagent and reacted at room temperature for 20 min. The absorbance was then determined with a multi-detector microplate reader VICTOR X3 (PerkinElmer, Waltham, MA, USA) at 540 nm using sodium nitrite as a standard.

### 4.8. Statistical Analysis

Each experiment was repeated more than three times. Results are presented as average ± standard deviation (mean ± SD). The significance of difference between mean values was determined with one-way analysis of variance (ANOVA) followed by Tukey’s test using GraphPad Prism 7 Software (San Diego, CA, USA).

## Figures and Tables

**Table 1:** List of primers for real-time RT-PCR.

Name	Forward (5′→3′) Reverse (5′→3′)
Filaggrin	CAC CGC GAT ACA GCC AGT AGC TGC CAT GTC TCC AAA CTA
Involucrin	GGG ACT GCC TGA GCA AGA AT GGA GCT CCA ACA GTT GCT CT
Loricurin	AAC AGT ATC AGT GCC AGA GC TCT GAC TGG TCT GCT GAG AG
Serine Palmitoyltransferase Long Chain Base Subunit 1 (SPTLC1)	GCG CGC TAC TTG GAG AAA GA TGT TCC ACC GTG ACC ACA AC
Serine Palmitoyltransferase Long Chain Base Subunit 2 (SPTLC2)	AGC CGC CAA AGT CCT TGA G CTT GTC CAG GTT TCC AAT TTC C
Thymus and activation-regulated chemokine (TARC)	CTT CTC TGC AGC ACA TCC AC CTG CCC TGC ACA GTT ACA AA
GAPDH	GTG GCA AAG TGG AGA TTG CC GAT GAT GAC CCG TTT GGC TCC
IL-6	TGG AGT CAC AGA AGG AGT GGC TAA TCT GAC CAC AGT GAG GAA TGT CCA C
β-actin	GAC AGG ATG CAG AAG GAG ATT ACT TGA TCC ACA TCT GCT GGA AGG T
